# Organization of Posterior Parietal–Frontal Connections in the Rat

**DOI:** 10.3389/fnsys.2019.00038

**Published:** 2019-08-21

**Authors:** Grethe M. Olsen, Karoline Hovde, Hideki Kondo, Teri Sakshaug, Hanna Haaland Sømme, Jonathan R. Whitlock, Menno P. Witter

**Affiliations:** The Faculty of Medicine, Kavli Institute for Systems Neuroscience, Centre for Neural Computation, Egil and Pauline Braathen and Fred Kavli Centre for Cortical Microcircuits, NTNU—Norwegian University of Science and Technology, Trondheim, Norway

**Keywords:** anterograde tracer injections, retrograde tracer injections, immunohistochemistry, cingulate cortex, motor cortex, orbitofrontal cortex, posterior parietal cortex

## Abstract

Recent investigations of the rat posterior parietal cortex (PPC) suggest that this region plays a central role in action control together with the frontal cortical areas. Posterior parietal-frontal cortical connections have been described in rats, but little is known about whether these connections are topographically organized as in the primate. Here, we injected retrograde and anterograde tracers into subdivisions of PPC as well as the frontal midline and orbital cortical areas to explore possible topographies within their connections. We found that PPC projects to several frontal cortical areas, largely reciprocating the densest input received from the same areas. All PPC subdivisions are strongly connected with the secondary motor cortex (M2) in a topographically organized manner. The medial subdivision (medial posterior parietal cortex, mPPC) has a dense reciprocal connection with the most caudal portion of M2 (cM2), whereas the lateral subdivision (lateral posterior parietal cortex, lPPC) and the caudolateral subdivision (PtP) are reciprocally connected with the intermediate rostrocaudal portion of M2 (iM2). Sparser reciprocal connections were seen with anterior cingulate area 24b. mPPC connects with rostral, and lPPC and PtP connect with caudal parts of 24b, respectively. There are virtually no connections with area 24a, nor with prelimbic or infralimbic cortex. PPC and orbitofrontal cortices are also connected, showing a gradient such that mPPC entertains reciprocal connections mainly with the ventral orbitofrontal cortex (OFC), whereas lPPC and PtP are preferentially connected with medial and central portions of ventrolateral OFC, respectively. Our results thus indicate that the connections of PPC with frontal cortices are organized in a topographical fashion, supporting functional heterogeneity within PPC and frontal cortices.

## Introduction

The posterior parietal cortex (PPC) is a multimodal association area, proposed to play a role in a variety of higher cognitive functions. In the rat, many functional studies of PPC have focused on its role in spatial navigation (Kolb and Walkey, 1987; Chen et al., 1994a,b; Save and Moghaddam, 1996; Save and Poucet, 2000; Save et al., 2005; Nitz, 2006, 2012). In contrast, in the non-human primate, the focus has been on a presumed function in action control and therefore on the interaction between PPC and frontal cortices (Cavada and Goldman-Rakic, 1989b; Andersen et al., 1990; Pesaran et al., 2008; Gharbawie et al., 2011; Stepniewska et al., 2011). In humans, the early exemplary role of parts of PPC were described in terms of contralateral neglect (Mesulam, 1999; Corbetta and Shulman, 2011) and, interestingly, comparable deficits have been reported in monkeys (Deuel and Regan, 1985) and rats (King and Corwin, 1993; Burcham et al., 1997).

Probing and comparing the functional relevance of PPC has been complicated by the fact that the physical location and delineation of PPC in different species is disputed, and an overall consensus on whether the PPC in different species actually is a homologous area is lacking (Olsen and Witter, 2016). For example, even within the rat, the delineation and functional division of PPC has been variable (for a review, see Whitlock et al., 2008). In a recent study, we defined PPC in the rat on the basis of a combination of cyto- and chemo-architectonic criteria and patterns of thalamic connectivity. This resulted in a reliable subdivision into three domains: a medial (mPPC), lateral (lPPC) and caudolateral (PtP) subdivision (Olsen and Witter, 2016). In the mouse, the position and definition of the main borders of PPC with its neighbors, the visual, somatosensory, and motor cortices and the subdivisions used here are comparable (Hovde et al., 2018), although some authors additionally differentiate nearby subareas in mice based on projections from primary visual cortex and their specific visual properties (areas RL, A, AM, Wang and Burkhalter, 2007; Wang et al., 2011).

Since early functional studies of the rat PPC focused on the role of this region in spatial navigation, we recently investigated the connections between PPC and the cortical regions most critical for such behavior, the hippocampal and parahippocampal regions, and found that this connectivity in general, was sparse, with the exception of projections to the presubiculum. We additionally described projections to retrosplenial cortex (RSC; Olsen et al., 2017). It is therefore unlikely that PPC provides a functional signal that is directly relevant for the emergence of spatially modulated neurons found in the hippocampal-projecting medial entorhinal cortex, such as grid cells, border cells, head direction cells or speed cells (Fyhn et al., 2004; Hafting et al., 2005; Sargolini et al., 2006; Solstad et al., 2008; Kropff et al., 2015). In parallel, there has been an increasing interest in the communication between PPC and frontal cortex in rodents (King and Corwin, 1992, 1993; Burcham et al., 1997; Erlich et al., 2015; Hanks et al., 2015), especially their respective roles in decision-making, which complements the long history of work on similar topics in primates. Recent work has also confirmed that similar projections link PPC and frontal cortical areas in mice (Hovde et al., 2018). However, in contrast to thorough analyses in the non-human primate, where these connections have been found to be topographically organized (Pandya and Kuypers, 1969; Petras, 1971; Mesulam et al., 1977; Cavada and Goldman-Rakic, 1989b; Andersen et al., 1990; Neal et al., 1990; Lewis and Van Essen, 2000; Rozzi et al., 2006), data on posterior parietal-frontal cortical connectivity in rodents is less detailed, confounding functional analyses of their interactions. Reciprocal connections of PPC with frontal midline and orbital cortices have been described (Reep et al., 1984, 1987, 1990, 1994, 1996; Kolb and Walkey, 1987; Condé et al., 1995; Hoover and Vertes, 2007, 2011), but to our knowledge no studies have systematically investigated the specificity of the organization of these connections, in particular how the three PPC subdivisions described previously (Olsen and Witter, 2016) relate to the frontal cortex. Thus, the present study aims to illuminate the topographical organization of the posterior parietal-frontal cortical connections in the rat.

## Materials and Methods

### Animals and Surgeries

All experimental procedures followed approved protocols that adhere to national and EU regulations. We analyzed 74 injections of anatomical tracers in 61 Sprague–Dawley rats (58 females, three males, 180–230 g at the time of surgery; Charles River, Sulzfeld/Kisslegg, Germany). The majority of the material (65 cases) described here was obtained in previous studies and methods for tracer injections, perfusions and histology are described in detail there (Kondo and Witter, 2014; Olsen and Witter, 2016). To complement the already existing material, a few cases were prepared with successful injections of anterograde tracers in the dorsolateral part of the orbitofrontal cortex (DLO; *N* = 1) and M2 (*N* = 3), as well as injections of retrograde tracers in the ventral and ventrolateral orbital cortex (VO/VLO region; *N* = 5).

In short, animals were deeply anesthetized and injected with retrograde and/or anterograde tracers in the parietal, orbitofrontal and medial frontal domains of the cortex. As retrograde tracers, we used Fast Blue (EMS Chemie, Domat/Ems, Switzerland, catalog number 9000002; 1% in 0.125 M phosphate buffer), and Fluorogold (Fluorochrome, Denver, CO, USA; 2.5% in H_2_O). For anterograde tracing, *Phaseolus vulgaris* Leucoagglutinin (PHA-L, Vector Laboratories, Burlingame, CA, USA, catalog number L-1110; 2.5% in 0.01 M phosphate buffer) and 10 kDa biotinylated dextran amine (BDA, Invitrogen, Molecular Probes, Eugene, OR, USA, catalog number D1956, RRID:AB_2307337; 5% solution in 0.125 M phosphate buffer) were used.

Rats were anesthetized with Isoflurane and injected i.p. with atropine (Nycomed, Zürich, Switzerland, 0.04 mg/kg) and rimadyl (Pfizer, New York, NY, USA, 5 mg/kg) and placed in a stereotaxic frame (Kopf Instruments, Tujunga, CA, USA). During surgery, we maintained a constant body temperature of 37°C. Stereotaxic coordinates were determined using Bregma and the mid-sagittal sinus as rostral-caudal and medial-lateral reference points, respectively, using a stereotaxic atlas as a guide (Paxinos and Watson, 2007). Retrograde tracers were pressure-injected into the brain through 1 μl Hamilton syringes. Iontophoretic injections of anterograde tracers were performed using glass micropipettes with an outer tip diameter of 15–25 μm (alternating currents, 6 s on/6 s off, 6 μA for BDA and 7 μA for PHA-L). During the surgery, the rat was given saline subcutaneously to avoid dehydration. Upon completion of injections, the wound was cleaned and sutured, and the animal was allowed to recover in a heat chamber before being returned to its home cage.

### Perfusion and Tissue Processing

After a survival time of 1–2 weeks to allow for complete transport of the tracers, the animals were anesthetized and transcardially perfused with Ringer solution (37°C) followed by freshly depolymerized 4% paraformaldehyde (pH 7.4). The brains were extracted and post-fixed in the perfusion fixative overnight. After being cryoprotected in a DMSO/glycerol solution at least overnight, six equally spaced series of 50 μm coronal sections were prepared on a freezing microtome. One series was mounted on Superfrost plus-slides and stained with cresyl violet for cytoarchitectural orientation. For brains containing fluorescent retrograde tracers, one series was mounted on uncoated microscope slides for analysis of labeling without any further processing, and one series of the brains containing anterograde tracers was processed to reveal the transport of BDA and PHA-L following standard (immuno)histochemical protocols for free-floating sections. Fluorescent molecules or the photostable molecule 3,3‣-diaminobenzidine tetrahydrochloride (DAB, Sigma-Aldrich, St. Louis, MO, USA; catalog number D5905) were used as chromophores (for further details see Olsen and Witter, 2016).

### Imaging and Analyses

In order to delineate the PPC, the frontal midline cortex, and the OFC, we used Nissl stained sections at appropriate levels. Images were obtained using a Mirax-midi scanner with a white light source (objective 20×, NA0.8; Carl Zeiss MicroImaging, Jena, Germany), or a Zeiss Axio Imager M2 microscope (Carl Zeiss MicroImaging). Using Adobe Photoshop CS6 (Adobe Systems Incorporated, San Jose, CA, USA), the images were converted to grayscale images, and the images were adjusted using the Levels function to improve the illustration of the cytoarchitecture of the cortical areas. Mainly, the black point was set to higher values whereas the gamma value was decreased to better visualize Nissl labeled cell bodies. The adjusted images were imported to Adobe Illustrator CS6 (Adobe Systems Incorporated), and borders of cortical areas were added.

Representative cases were selected for illustration of labeling patterns. For all tracer injections, labeling patterns are shown in the ipsilateral hemisphere, and for illustration purposes, all injections are shown as being in the right hemisphere although a few were in the left hemisphere. Retrogradely labeled cell bodies were mapped using a microscope connected to a computer with Neurolucida software (MicroBrightField, Colchester, VT, USA). The resulting maps were overlaid with images of adjacent Nissl-stained sections in Adobe Illustrator CS6 and the regions of interest were delineated. To illustrate anterogradely labeled fibers, images were obtained using a Mirax-midi scanner with a fluorescent or white light source, or a Zeiss Axio Imager M2 microscope. Pictures of DAB labeled fibers were turned into pseudo-dark-field images or grayscale images, whereas images of fluorescent labeled fibers were exported as grayscale images. Brightness and contrast were adjusted in Adobe Photoshop CS6 to improve visualization of the labeled fibers. Due to the generally dim nature of the images, the white point was shifted to lower values and the gamma value was increased. The position of labeled elements was determined using adjacent Nissl stained sections from the same brains allowing for the delineation of regions of interest with the use of Adobe Illustrator CS6.

### Flatmap Illustrations

To illustrate connections between PPC and frontal midline cortex, three different representations were used. First, we illustrated the location and density of labeled neurons and fibers in actual histological sections, either as drawings or as images. Second, a grayscale table was produced for each representative case of retrograde tracer injected in PPC. To this end, every section along the rostrocaudal axis of a specific cortical area was delineated, and the density of retrogradely labeled cells was scored subjectively for each cortical area with the densest labeling in each experiment being indicated by the darkest gray. The results were plotted in an Excel table, where each column represents a coronal section and each row represents a cortical area in that section. Third, standardized representations of the location of projections/labeled fibers from each subregion of PPC to the superficial layers of frontal midline cortex were produced. These normalized “flatmaps” contained the average of projections across five cases for each PPC subregion. Seven cases were excluded from this analysis due to tilted cutting angle, poor images or missing tissue. However, the overall labeling in the excluded brains was the same as for the included cases. To create the flatmap representations, every section along the rostrocaudal axis of areas 24a, 24b, M2 and M1 was delineated. The distance from the medial border to labeled fibers was measured through layer 2/3, and the density of labeling was subjectively scored on a scale from 0 to 5 for each cortical area, 0 indicating no labeling. Further, the scale was normalized for each brain such that “5” corresponded to the strongest labeling in M2 in that brain, since M2 contained the heaviest labeling in all cases. The sections across brains were aligned using the level at which the forceps minor, the genu of corpus callosum and CA3 appeared in coronal sections. The sections were spaced 0.3 mm apart and assigned corresponding Bregma levels that were then used by a custom-made Matlab script to generate the flatmaps as described in detail below.

#### Individual Flatmaps

Given that the thickness of each brain section was 50 μm and the distance between brain sections was slightly variable, the intermediate values for the total lengths of the region borders were linearly interpolated using 50 μm intervals. In order to use an equilateral square pixel as the basic unit of the reconstructed flatmap of the regions, the total lengths at each point in the rostro-caudal axis were divided by 50 μm units. The intermediary product, the region outline flatmap, consisted of a row of contiguous columns of pixels, with the number of pixels in each column given by the total length of that region at that rostrocaudal coordinate. Next, the region outline flatmap was filled with the values of labeled fiber densities. Taking into consideration that the density values were registered at non-contiguous points in the rostrocaudal axis of the flatmap, it was necessary to assign them at the corresponding columns in the map and interpolate the values of the columns in between. For the columns where labeled fiber intensity data were available, the partial lengths for each block were divided by 50 μm units, and rounded to the nearest integer, which gave the number of pixels that was assigned to the corresponding value of intensity measured. The blocks were assigned starting at the unit nearest to the region’s 24b/M2 border and moving laterally. Once the assigned columns were filled, a three-step process was used to fill the intermediary columns. (1) For each pair of consecutively filled columns, the length of the longest column of the two was taken as reference. The shortest of the two columns was then stretched to the same length as the reference, using a nearest neighbor interpolation algorithm with rounding to the nearest integer, such that the proportional size of the blocks was preserved in the stretched column. This step was tested with examples in order to assure that the process is faithful during reversal, i.e., the block distribution is similar to the original when the column is set back to its original length. When both columns were adjusted to the same length, they were set as the first and the last column in a rectangle. This rectangle width was dependent on the distance between two consecutive filled sections, divided by the lateral length of the unitary pixel (50 μm). (2) A linear interpolation algorithm was used to fill the intermediary pixels on each row of the rectangle, followed by rounding each pixel to the nearest integer. (3) Every column of the group being processed was adjusted back to the length value calculated previously for the corresponding position of the rostrocaudal axis in the region outline flatmap. This step was achieved using the same nearest neighbor interpolation algorithm, with rounding to the nearest integer that was used on the first step of the process. This process was repeated for all pairs of consecutively filled columns. At the end of this iterative process, a region flatmap with continuous values of labeled fiber density had been created. The four regions (regions 24a, 24b, M2 and M1) were assembled into a final flatmap. This was done by iteratively joining the rostrocaudal coordinates of the corresponding columns of each of the individual flatmaps, starting at the most medial region at the bottom and proceeding through all the regions to the most lateral at the top. White lines were plotted at the junction points in order to visually separate the four regions. Regions with the densest labeling in each experiment are indicated by the brightest color. For individual maps, see Supplementary Figure S1.

Two important arbitrary aspects were assumed in order to calculate the average flatmaps. The first was that the process of averaging was done individually for every region, and the final result was the product of assembling the four region maps together. The second was that the areas of the average region maps did not reflect the mean of the areas of all the individual maps. Rather, the largest of all the individual maps was used as reference and the data from the smaller maps was stretched to its size. This was done because stretching and interpolating a set of two-dimensional data does not lead to loss of data, whereas the opposite process can. Another reason was that there were small variations in the shapes of the individual maps, so by doing this, the average flatmaps were close to the ones of their origin. The algorithm used is similar to that used for flatmaps in Sugar and Witter (2016), where a graphical representation of the algorithm is provided.

## Results

### Topography and Delineations of Cortical Areas

**Figure 1:**
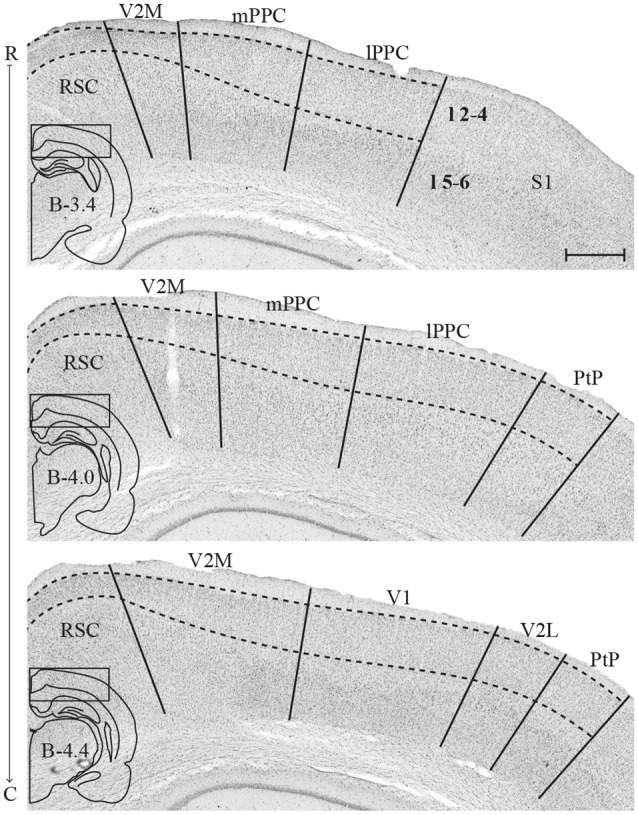
Delineation of posterior parietal cortex (PPC) and adjacent areas. Coronal sections containing PPC, arranged from rostral to caudal (indicated as R–C). Solid lines indicate borders between cortical areas and dashed lines demarcate cortical layers. Insets outline the hemisphere of the depicted coronal section and the area shown. Bregma levels are approximate and according to Paxinos and Watson (2007). Scale bar: 500 μm.

The PPC in the rat is situated dorsally in the brain, between the somatosensory parietal and visual occipital domains, and comprises three subdivisions, the medial (mPPC), lateral (lPPC) and the caudolateral (PtP) PPC (Figure 1; Olsen and Witter, 2016). mPPC is characterized by a homogenous appearance, whereas lPPC is perceived as slightly more laminated due to a sparsely populated layer 5. A defining feature of area PtP is the small and weakly stained cells of layer 3/4.

**Figure 2:**
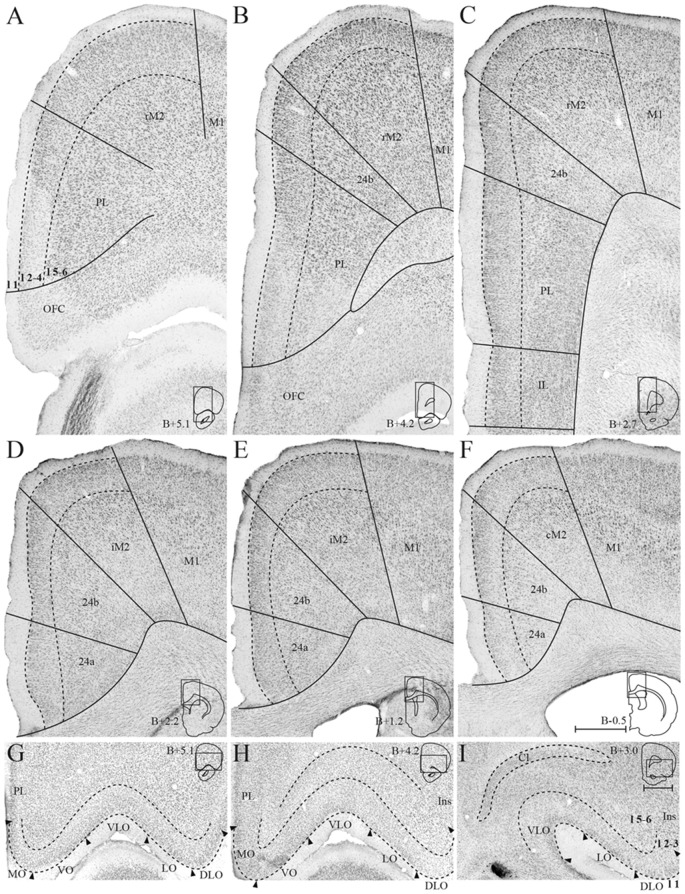
Delineation of the frontal midline and orbitofrontal cortices. Coronal sections containing the frontal midline cortex **(A–F)** and the orbitofrontal cortex (OFC; **G–I**), arranged from rostral to caudal. Solid lines **(A–F)** and black arrowheads **(G–I)** indicate borders between cortical areas and dashed lines demarcate cortical layers. Insets on the right outline the hemisphere of the depicted coronal section and the area shown. Bregma levels are approximate and according to Paxinos and Watson (2007). Scale bar: 500 μm.

The medial surface of the rat frontal cortex comprises several distinct areas (Figure 2; Jones et al., 2005; Vogt and Paxinos, 2014). At rostral levels, prelimbic cortex (PL) is found ventral to the rostral secondary motor cortex (rM2) and dorsal to orbitofrontal cortex (OFC). Throughout most of its rostrocaudal extent, M2 is bordered laterally by primary motor cortex (M1). Approximately at the level of the forceps minor of the corpus callosum, the anterior cingulate cortical (ACC) area 24b is wedged between PL and rM2, and infralimbic cortex (IL) is seen ventral to PL (Figure 2B). At the level of the genu of the corpus callosum, IL and PL disappear and ACC area 24a is situated between the corpus callosum and area 24b (Figure 2D). Caudally, at the level where the hippocampus appears, areas 24a and 24b are replaced by retrosplenial cortex (RSC, not shown). The IL is the least differentiated of the frontal midline areas, it is poorly laminated and the border between layers 1 and 2/3 is particularly irregular. The dorsally adjacent PL is characterized by large, darkly stained cells in layer 5 and densely packed cells in layer 2. In area 24b, dorsal to PL in rostral sections, cells in layer 2 are darkly stained. Layer 5 is broad and contains a large number of pyramidal neurons. In area 24a, ventral to 24b at more caudal levels, cells are distributed homogenously across layers and 24a thus appears less laminated than 24b. In addition, superficial cells have larger somata in area 24a than in area 24b. Lateral to 24b, M2 comprises narrow superficial layers where layer 3 is weakly stained, and layer 5 appears homogenous and densely packed. On the basis of differences in connectivity, M2 has been divided into three parts along its rostrocaudal axis, the rostral part (rM2) being situated rostral to the genu of the corpus callosum, the intermediate part extending from the genu to the anterior commissure (iM2), and the caudal M2 (cM2) that extends from the anterior commissure until it is replaced caudally by the medial secondary visual cortex (Reep et al., 1990; Olsen and Witter, 2016). Similarly, area 24b has been hypothesized to contain three rostrocaudal divisions, of which the rostral portion is situated rostral to the genu of the corpus callosum (Jones et al., 2005).

**Figure 3:**
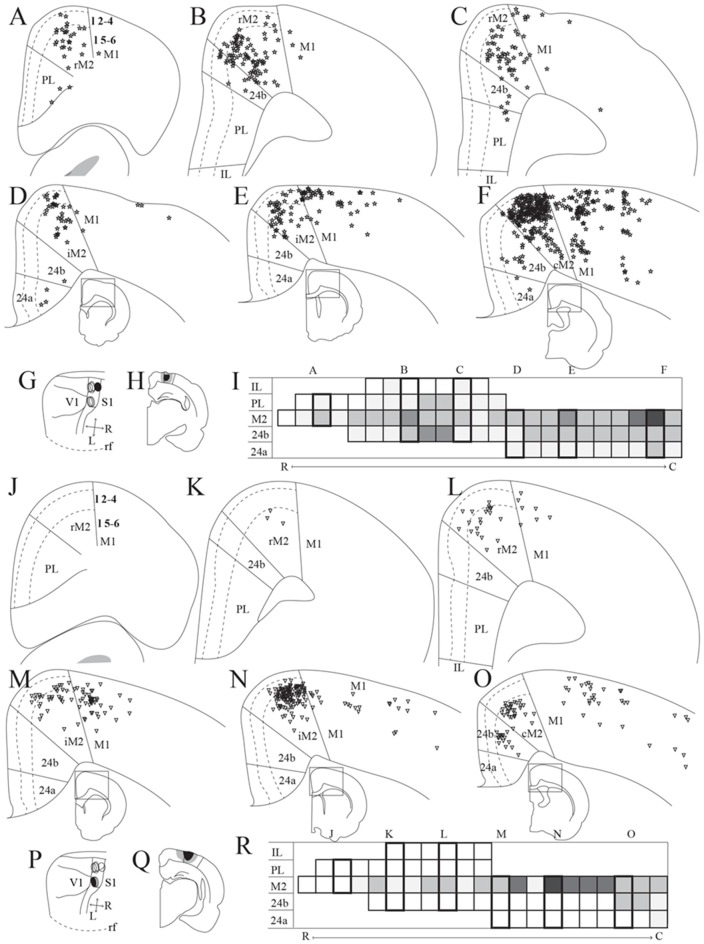
Frontal midline cortical input to PPC. Retrogradely labeled cells in the frontal midline cortex resulting from an injection of Fast Blue in medial PPC (mPPC; **A–F**, labeled cells represented with stars) and Fluorogold in lateral PPC (lPPC; **J–O**, labeled cells represented with triangles). **(A–F)** and **(J–O)** Coronal sections arranged from rostral **(A,J)** to caudal **(F,O)**. Solid lines indicate borders between cortical areas and dashed lines demarcate cortical layers. Insets in panels **(D–F)** and **(M–O)** indicate the rostrocaudal position of the section and the approximate area shown. Densest labeling was seen in area M2 throughout the rostrocaudal axis for both injections. **(G,P)** The respective injection sites represented in a surface rendering of the brain.** (H,Q)** Drawings of the respective injection sites in a coronal section of the brain. **(I,R)** Grayscale representation of the density of labeling in frontal midline areas from rostral to caudal (R–C), darker gray indicates a denser cluster of labeled cells. Each row represents a cortical area and columns represent coronal sections along the rostrocaudal axis. Columns representing sections visualized in the figure are boxed and indicated by their appropriate letter.

The OFC is situated ventrally and rostrally in the rat brain, on the dorsal bank of the rostral extension of the rhinal fissure. From medial to lateral, OFC comprises a medial (MO), ventral (VO), ventrolateral (VLO), lateral (LO), and dorsolateral (DLO) subdivision (Figure 3; Kondo and Witter, 2014). MO and VO constitute the medial bank of the rhinal fissure, VLO sits around the notch of the fissure, and LO and DLO are situated on the lateral bank. MO is bordered dorsally by PL at rostral levels and IL at caudal levels, whereas the insular cortex constitutes the dorsolateral border of DLO. OFC subdivisions are most easily distinguished based on the morphology of their superficial layers, whereas deep layers of this cortex are more homogenous and thus difficult to separate. MO layer 2 is sparsely populated with patches of cells, and the transition between layers 2 and 3 is diffuse. In the laterally adjacent area VO, superficial layers contain smaller cells than in MO. VO is overall more sparsely populated than its neighboring areas, giving it a homogenous appearance. VLO is densely packed with cells across layers and is particularly characterized by columns of cells in layer 2, organized perpendicular to the pia. LO layer 2 contains large, clustered cells, whereas layer 3 cells are small and densely clustered. DLO is distinguished from LO by comparatively larger cells in layer 3.

### Characterization of Injection Sites

Tracer injections in the PPC were described in detail in a previous study where the positions of the injections were characterized based on the cytoarchitecture of the area as well as the resulting thalamic labeling pattern (Olsen and Witter, 2016). A surface representation of the cores of the retrograde and anterograde injection sites is given in Figures 3G,P and Figures 5–7G, respectively. In general, retrograde tracer injections tended to diffuse more and were more likely to extend into adjacent cortical areas compared to anterograde tracer injections.

**Figure 4:**
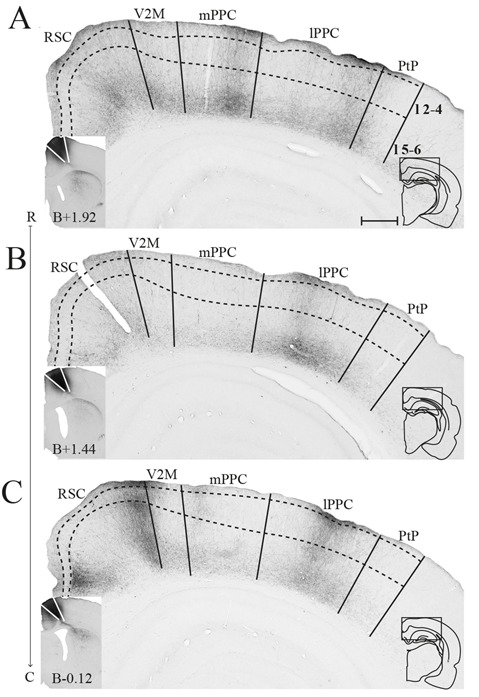
M2 projections to PPC. Anterogradely labeled fibers in PPC and nearby areas resulting from three injections of PHA-L, each at a different rostrocaudal level of M2 (indicated by R–C). Although the cores of the injections were located at different levels, all injections extended in the rostrocaudal axis and partially overlapped. The most rostral injection in iM2 **(A)** resulted in dense plexuses of labeled fibers in mPPC and lPPC, whereas the more caudal injections in iM2 **(B)** and at the border between iM2 and cM2 **(C)** resulted in densest labeling in lPPC. Solid lines indicate borders between cortical areas and dashed lines demarcate cortical layers. Insets are images of the core of the injection sites with the borders of M2 indicated by solid lines (left) and outlines of the hemisphere of the depicted section and the area shown containing labeled fibers (right). Bregma levels are approximate and according to Paxinos and Watson (2007). Scale bar: 500 μm.

**Figure 5:**
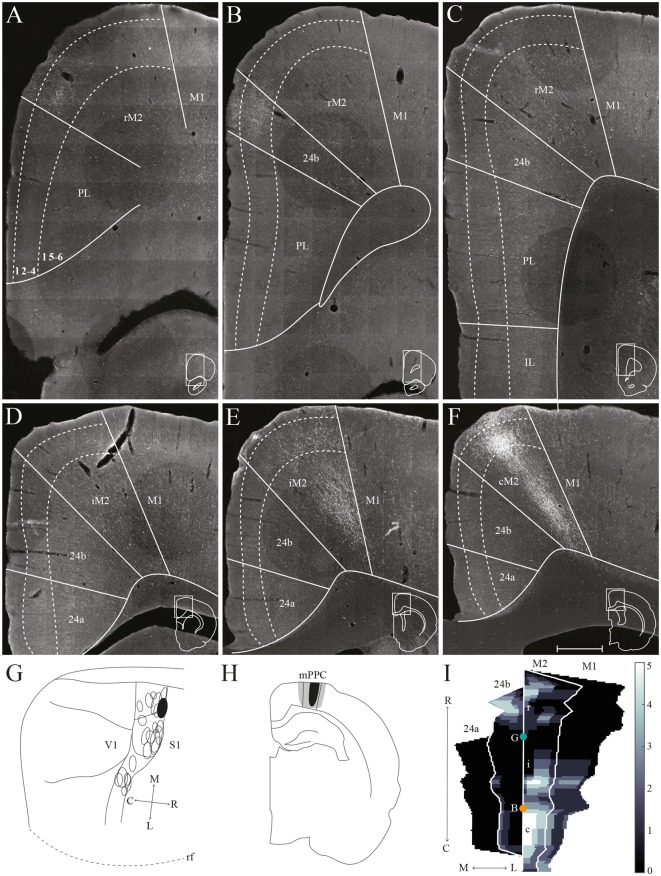
mPPC projections to frontal midline cortex. Anterogradely labeled fibers in the frontal midline cortex resulting from an injection of PHA-L in mPPC. **(A–F)** Coronal sections arranged from rostral **(A)** to caudal **(F)**. Solid lines indicate borders between cortical areas and dashed lines demarcate cortical layers. Insets in all panels indicate the rostrocaudal position of the section and the approximate area shown. Densest labeling was seen in area cM2. **(G)** PHA-L injection site in mPPC shown as a filled circle in a surface rendering of the brain and all injection sites. **(H)** Drawing of the injection site in a coronal section with the borders of mPPC indicated by solid lines. **(I)** Intensity representation of the density of labeling in frontal midline areas from rostral to caudal (R–C), Lighter color indicates a denser plexus of labeled fibers. The scale bar relates the color code in the flatmaps to the density scores initially assigned to each pixel in the digitized sections (for details, see “Materials and Methods” section). Bregma is indicated by an orange dot and the genu of the corpus callosum, which coincides with the border between rM2 and iM2, by a green dot. Scale bar: 500 μm.

**Figure 6:**
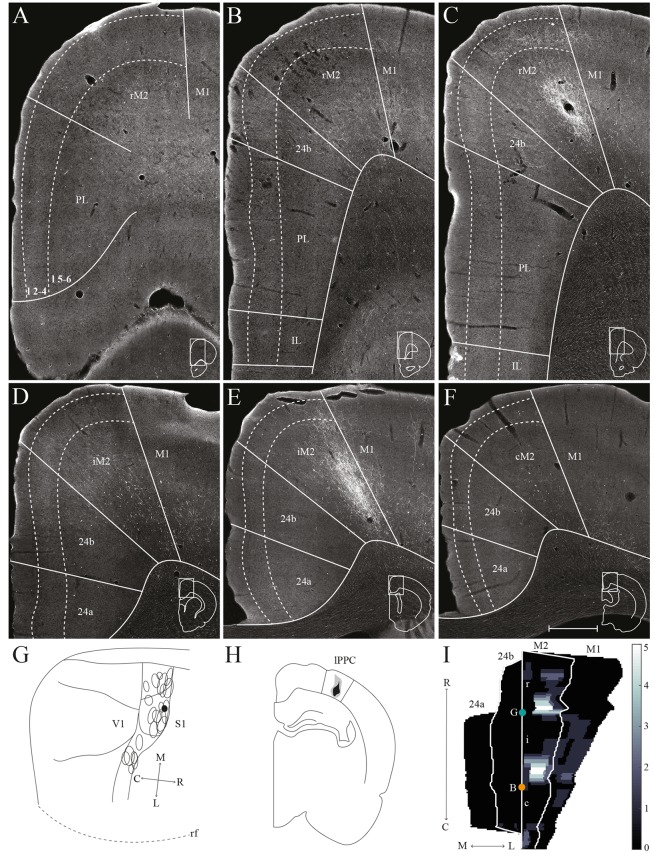
lPPC projections to frontal midline cortex. Anterogradely labeled fibers in the frontal midline cortex resulting from an injection of BDA in lPPC. **(A–F)** Coronal sections arranged from rostral **(A)** to caudal **(F)**. Solid lines indicate borders between cortical areas and dashed lines demarcate cortical layers. Insets in all panels indicate the rostrocaudal position of the section and the approximate area shown. Densest labeling was seen in area iM2. **(G)** BDA injection site in lPPC shown as a filled circle in a surface rendering of the brain and all injection sites. **(H)** Drawing of the injection site in a coronal section with the borders of lPPC indicated by solid lines. **(I)** Intensity representation of the density of labeling in frontal midline areas from rostral to caudal (R–C), lighter color indicates a denser plexus of labeled fibers. The scale bar relates the color code in the flatmaps to the density scores initially assigned to each pixel in the digitized sections (for details, see “Materials and Methods” section). Bregma is indicated by an orange dot and the genu of the corpus callosum, which coincides with the border between rM2 and iM2, by a green dot. Scale bar: 500 μm.

**Figure 7:**
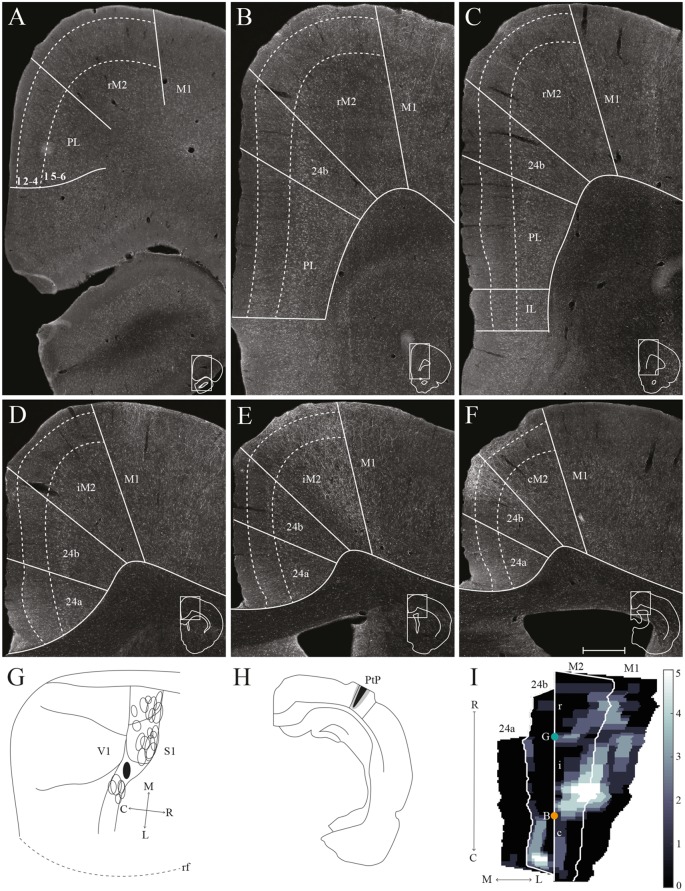
PtP projections to frontal midline cortex. Anterogradely labeled fibers in the frontal midline cortex resulting from an injection of PHA-L in PtP. **(A–F)** Coronal sections arranged from rostral **(A)** to caudal **(F)**. Solid lines indicate borders between cortical areas and dashed lines demarcate cortical layers. Insets in all panels indicate the rostrocaudal position of the section and the approximate area shown. Densest labeling was seen in area iM2. **(G)** PHA-L injection site in PtP shown as a filled circle in a surface rendering of the brain and all injection sites. **(H)** Drawing of the injection site in a coronal section with the borders of PtP indicated by solid lines. **(I)** Intensity representation of the density of labeling in frontal midline areas from rostral to caudal (R–C), lighter color indicates a denser plexus of labeled fibers. The scale bar relates the color code in the flatmaps to the density scores initially assigned to each pixel in the digitized sections (for details, see “Materials and Methods” section). Bregma is indicated by an orange dot and the genu of the corpus callosum, which coincides with the border between rM2 and iM2, by a green dot. Scale bar: 500 μm.

Injections of anterograde tracers in MO, VO, VLO and LO of OFC were part of a previous study, in which the injections were described extensively (Kondo and Witter, 2014). However, the latter study did not include tracer injections in DLO, nor did it include injections of retrograde tracers in OFC. Therefore, we supplemented the material with one injection of anterograde tracer in DLO (not shown) as well as five injections of retrograde tracers in the VO/VLO areas. In addition to the surface representations of the injection sites illustrated here and in previous studies, all illustrations of labeled neurons or fibers in the present study include a representation of the core of the tracer injection site as seen in a coronal section.

### Connections of PPC with Frontal Midline Areas

#### Input to PPC From Frontal Midline Areas

We analyzed seven injections of retrograde tracers in PPC, four in mPPC and three in lPPC, since we, unfortunately, did not obtain selective injections of retrograde tracers in PtP. All injections resulted in highest densities of labeled cells in M2, with less labeling in other frontal midline areas (mPPC; Figures 3A–F,I, lPPC; Figures 3J–O,P). In a representative case, Fast Blue was injected rostrally in mPPC (Figures 3G,H). Throughout the rostrocaudal length of the frontal midline cortex, retrogradely labeled cells were seen in M2 (Figures 3A–F,I), though labeling was especially dense in the most caudal parts (Figure 3F). We observed labeled neurons across all layers but with a denser concentration in layer 5. Retrogradely labeled cells were also found in area 24b but in much lower numbers than in M2 (Figures 3B–F). Occasionally labeled cells were encountered in PL and 24a (Figures 3A,C,D,F).

Retrograde tracer injections in lPPC also resulted in retrogradely labeled cells mainly in M2, showing a slightly different pattern than seen in mPPC cases. In a representative case, Fluorogold was injected caudally in lPPC on the border with mPPC and impinging on visual areas (Figures 3P,Q). Retrogradely labeled cells were found almost throughout the rostrocaudal extent of M2 (Figures 3J–O,R) with the exception of the part rostral to the forceps minor of the corpus callosum, where only low numbers of neurons were labeled (Figures 3J,K,O). Similar to mPPC cases, the majority of the labeled cells were encountered in layer 5, but in contrast, their rostrocaudal position was different such that the densest M2 labeling was concentrated at a slightly more rostral level in the lPPC cases compared to the labeling seen in mPPC cases (Figures 3I,R). In area 24b labeled cells were found only at the most caudal levels and no labeled cells were observed in PL, IL or 24a (Figures 3J–O,R).

In three animals, PHA-L was injected in M2 (Figure 4). Although the cores of the injections were at different rostrocaudal levels, all injections extended along the rostrocaudal axis and partially overlapped. All injections covered deep layers, which in our retrograde data were shown to be the main origin of projections to PPC, whereas involvement of superficial layers was more variable between cases. Although the injections were largely confined to M2, they did impinge on the medially adjacent 24b and laterally adjacent M1. All three cases resulted in dense labeling in PPC. In the case with the most rostrally located injection in iM2, one dense plexus of labeled fibers were found in mPPC and another in lPPC (Figure 4A). Labeling was particularly dense in layers 1 and 6, where the fibers appeared to branch strongly, indicating that this was where the fibers terminated. Fibers going through other layers were largely straight with minimal branching but did show swellings and thus showed a beaded morphology. These are believed to represent mitochondria although en passant synapses cannot be excluded. In the two cases with more caudally positioned injections, in iM2 and on the border between iM2 and cM2, sparser labeling was seen in mPPC and a dense plexus of labeled fibers spanning across layers was observed in lPPC (Figures 4B,C). Similar to the results from the more rostral case with the injection located in M2, terminating fibers were particularly focused in layers 1 and 6 of PPC. In all three cases, labeling was sparser within area PtP than in mPPC and lPPC. Also, in all three cases, labeled fibers were observed in PPC of the hemisphere contralateral to the injection, but the density was drastically reduced. Similar to the ipsilateral labeling, labeled contralateral fibers terminated mainly in layers 1 and 6 although sparse plexuses of labeled fibers were seen stretching across the cortical layers at positions homotopic to the ipsilateral plexuses (Supplementary Figure S2).

#### PPC Projections to Frontal Midline Areas

**Figure 8:**
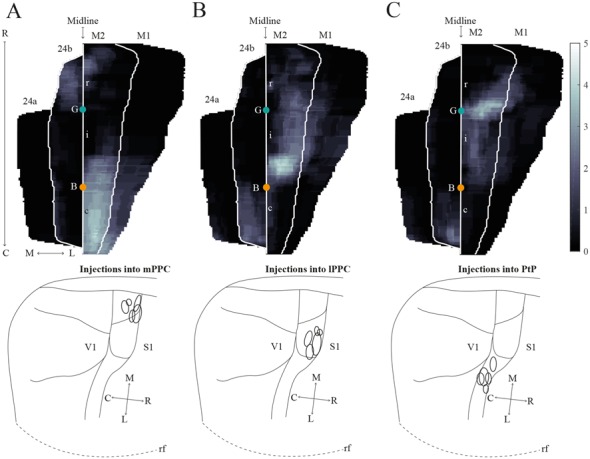
Average projections from PPC to midline frontal cortices. **(A–C)** Top: average intensity representation of the density of labeling in frontal midline areas from rostral to caudal (R–C), resulting from injections of anterograde tracers into mPPC **(A)**, lPPC **(B)**, and PtP **(C)**. Lighter color indicates a denser plexus of labeled fibers. The scale bar relates the color code in the flatmaps to the density scores initially assigned to each pixel in the digitized sections (for details, see “Materials and Methods” section). Bregma is indicated by an orange dot and the genu of the corpus callosum, which coincides with the border between rM2 and iM2, by a green dot. Bottom: surface representations of injection sites in cases that make up the average map.

We analyzed 22 injections of anterograde tracers in PPC, seven in mPPC, nine in lPPC and six in PtP. Injections of anterograde tracers in PPC resulted in dense anterograde labeling in M2, with less labeling in other frontal midline areas (Figures 5–8).

##### mPPC

In a representative case, PHA-L was injected rostrally in mPPC, covering all layers (Figures 5G,H). In the most rostral parts of area rM2, sparse terminal labeling was present in superficial layers (Figure 5A). More caudally, labeling was denser and present across layers (Figure 5E). The densest labeling was seen in cM2 (Figures 5F,I), where terminal fibers branched strongly in layers 1, 3, and deep 5. It is worth noting that this densely labeled plexus appears to be located at approximately the same rostrocaudal position as the dense patch of labeled cells after injecting a retrograde tracer in mPPC (Figure 3F). In area 24b, a restricted plexus of terminating fibers was found in superficial layers at its most rostral level (Figures 5B,I) and sparse labeling was observed in superficial and deep layers at the most caudal level of area 24b (Figures 5E,F,I), extending ventrally into area 24a and caudally into RSC (not shown). No labeled fibers were encountered in IL, and only a few were observed in PL (Figures 5A–C). In the contralateral hemisphere, sparse labeling reflected the ipsilateral labeling and was most frequently found in M2 at the same rostrocaudal levels as the strongest ipsilateral labeling (not shown). The majority of the contralateral labeled fibers were located in layers 1 and 3, and comparably fewer fibers were observed in layer 5. Other cases of tracer injections in mPPC yielded similar labeling patterns. In several cases, sparse to moderate labeling was observed in rM2 rostral to the forceps minor, with some fibers also located in area 24b, and comparably less labeling was found in iM2. The densest labeling was most frequently found caudal to the crossing of the anterior commissure although the exact rostrocaudal level of the densest plexus could vary between cases.

##### lPPC

A representative, small BDA injection in lPPC with a core mainly in deep layers was centered at a mid rostrocaudal level (Figures 6G,H). Similar to the results in case of the injection in mPPC, dense labeling was observed in M2, however, the labeling was shifted along the rostrocaudal axis (Figures 6C–E,I). No labeling was encountered at the most rostral levels (Figures 6A,I), and only sparse labeling was observed in rM2 at the level of the forceps minor of the corpus callosum (Figures 6B,I). A moderately dense plexus of terminal fibers in layers 3 and 5 were present rostral to the level of the genu of the corpus callosum, with fewer fibers in layers 1 and 6 (Figures 6C,I). At more caudal levels, another moderately dense plexus of terminating fibers was seen in iM2 with a similar laminar pattern (Figures 6E,I). This plexus was located at a similar rostrocaudal level as the dense patch of labeled cells seen after retrograde tracer injections in lPPC (Figures 3N,R), and thus at a slightly more rostral level compared to the densest plexus of labeled fibers in the above-described mPPC case. Labeled fibers were occasionally observed in area 24b, at the most caudal level they extended into area 24a (Figures 6F,I) and extended caudally into RSC (not shown). No labeled fibers were present in areas PL or IL (Figures 6A–C). Only a few labeled fibers were observed contralaterally, mainly in layer 5 of iM2 (not shown). Other cases with injections in lPPC produced similar labeling patterns. In all cases, although the exact rostrocaudal position varied slightly, the densest labeling in M2 was found in iM2. In addition, injections involving more superficial layers resulted in relatively more labeling in superficial layers of M2.

##### PtP

Similar to tracer injections in mPPC and lPPC, injections of anterograde tracers into PtP resulted in labeled fibers in M2. Following a representative injection impinging on the lateral secondary visual cortex (V2L, Figures 7G,H), very few labeled fibers were observed in rM2 at levels rostral to the genu of the corpus callosum (Figures 7A–C,I). More caudally, a dense plexus of labeled fibers were seen in area iM2, terminating mainly in layers 1, 3, and 5 (Figures 7E,I). This plexus appeared at a level similar to the dense plexus of labeled fibers that was seen after injecting tracer in lPPC (Figures 6E,I). At the most caudal level of the frontal midline cortex, a small but dense plexus of terminal fibers was found in superficial layers of area 24b with a few fibers extending into area 24a (Figures 7E,F,I) and continuing caudally into RSC (not shown). No labeled fibers were seen in areas PL or IL (Figures 7A–C). Very sparse labeling was seen in the contralateral hemisphere, homotopic to the densest ipsilateral labeling in M2 and 24b. Other cases of anterograde tracer injections into PtP yielded less labeling in M2, and although the densest labeling was consistently found within the iM2, labeling in several cases was shifted more rostrally, closer to the genu of the corpus callosum. Most cases had sparse labeling at caudal levels of area 24b/a continuing into RSC, however, two cases failed to yield labeling in area 24. In both cases, the injection was focused in layer 6 and deep layer 5 and overall cortical labeling was sparse.

In order to verify that the projection patterns presented for individual cases were representative of all animals, standardized representations of the location of labeled fibers in cingulate and motor areas were made in the form of flatmaps. Note that such flatmaps were made to illustrate and compare the preferred location of projections as well as relative strength. In each case, strength throughout the flatmap was represented in relation to the strongest labeling in M2, which was represented by a value of 5. The flatmaps were created by averaging the projection pattern across five brains with injections into mPPC (Figure 8A), five brains with injections into lPPC (Figure 8B) and five brains with injections into PtP (Figure 8C). For individual case maps see Supplementary Figure S1. The flatmaps confirmed the results from the representative cases and showed that mPPC, lPPC and PtP preferentially target different rostrocaudal portions of cingulate and motor cortices. mPPC preferentially targets cM2, with the strongest projections caudal to Bregma (Figure 8A). M2 labeling is accompanied by labeling in M1, which is strongest most caudally and tapers off rostral to Bregma. mPPC also has moderate projections targeting the most medial rM2, as well as to rostromedial 24b. Weak labeling was found on the medial border of 24b extending from the genu of the corpus callosum to the caudal extent of 24b. lPPC, in contrast, preferentially targets iM2 with the strongest projection observed in sections just rostral to Bregma, with moderate labeling continuing rostral and laterally in M2 (Figure 8B). Weaker projections are also seen medially in cM2, caudal to Bregma. Area 24b is labeled at the level of Bregma, with labeling extending caudally to where retrosplenial cortex appears. lPPC projections to M1 are weaker than to iM2 and 24b and target the mid rostrocaudal portion along the border of iM2. PtP also targets the iM2, though more weakly than lPPC, while its strongest projections are to more rostral levels near the genu of corpus callosum (Figure 8C). PtP projects weakly to caudal 24b, mainly terminating lateral to the projections from lPPC. Very weak labeling was also observed in caudal 24a for both lPPC and PtP injections.

### Connections of PPC With Orbitofrontal Cortex

#### Orbitofrontal Input to PPC

To analyze the termination pattern of orbitofrontal input to PPC, we analyzed our dataset of 36 anterograde tracer injections (Kondo and Witter, 2014), supplemented with one injection in DLO. Anterograde tracer injections in LO and DLO did not yield labeled fibers in PPC.

#### MO

**Figure 9:**
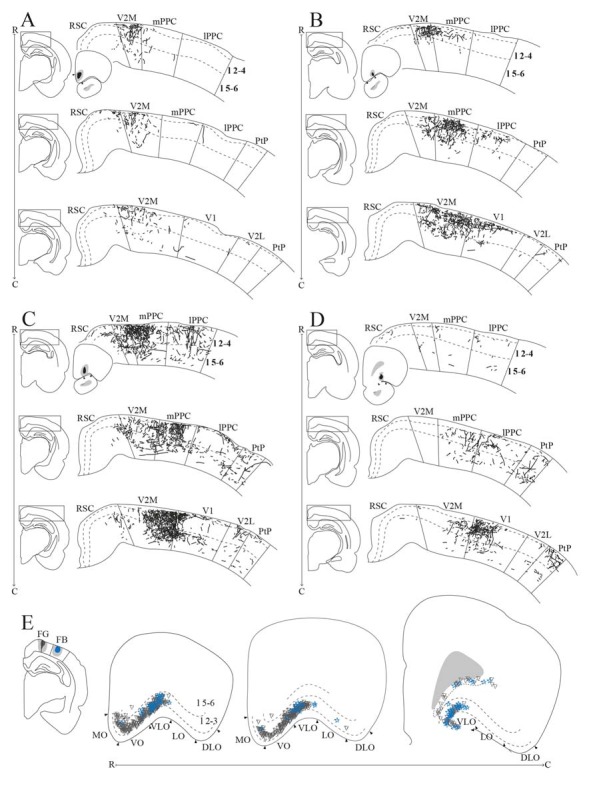
OFC projections to PPC. **(A–D)** Anterogradely labeled fibers at three different rostrocaudal levels of PPC (R–C, respectively) and adjacent areas resulting from injections of anterograde tracers in MO (**A**, BDA), VO (**B**, PHA-L), rostral VLO (**C**, PHA-L), and caudal VLO (**D**, BDA). As injection sites shifted from medial to lateral and caudal within OFC, anterogradely labeled fibers extended progressively more lateral and caudal in PPC. Solid lines indicate borders between cortical areas and dashed lines demarcate cortical layers. Right insets indicate the position of the core of the injection and the borders of MO **(A)**, VO **(B)**, and VLO **(C,D)** are marked by arrowheads. Left insets indicate the rostrocaudal position of the depicted area. **(E)** Retrogradely labeled cells in OFC, represented at three different rostrocaudal levels (indicated by R–C), resulting from an injection of Fluorogold in mPPC (represented by gray triangles) and injection of Fast Blue in lPPC (represented by blue stars) in the same animal. Both injections resulted in dense labeling in VLO, and the Fluorogold injection also resulted in dense labeling in VO and MO. Arrowheads indicate borders between cortical areas and dashed lines demarcate cortical layers. Inset indicates the position of the cores of the injection sites, with borders of mPPC and lPPC outlined with solid lines.

We analyzed seven anterograde tracer injections in MO, out of which only one injection of BDA yielded anterogradely labeled fibers in PPC (Figure 9A). This injection involved all layers and covered a large portion of the rostrocaudal extent of MO. The resulting labeling in PPC was sparse and mainly confined to mPPC, with only occasional labeled fibers in lPPC. No labeled fibers were encountered in PtP, whereas weak labeling was observed in the medial secondary visual cortex (V2M).

#### VO

Seven out of 11 anterograde tracer injections in VO resulted in labeled fibers in PPC. Generally, tracer injections situated medially within VO yielded less robust labeling in PPC than injections located more laterally in this area. In one representative case, PHA-L was injected laterally in VO on the border with VLO, where the core of the injection was confined to superficial layers (Figure 9B). The injection site slightly involved the anterior olfactory nucleus, which according to our retrograde data does not project to PPC. In this representative case, a moderately dense plexus of terminal fibers was observed in mPPC, sparser labeling was seen in lPPC, and only a few labeled fibers were encountered in PtP (Figure 9B). V2M, as well as primary visual cortex (V1), contained moderate labeling. In all areas, the majority of the labeled fibers were located in layers 2 and 3, whereas sparser labeling was seen in layers 1 and 5. Following a BDA injection in VO at the border with VLO, a moderately dense cluster of terminating fibers was located medially in mPPC, with clearly branching fibers in layers 2, 3, and 5 (see Figure 11A for exemplary micrograph). Only a few scattered labeled fibers were observed in lPPC, whereas in PtP labeling was absent.

**Figure 10:**
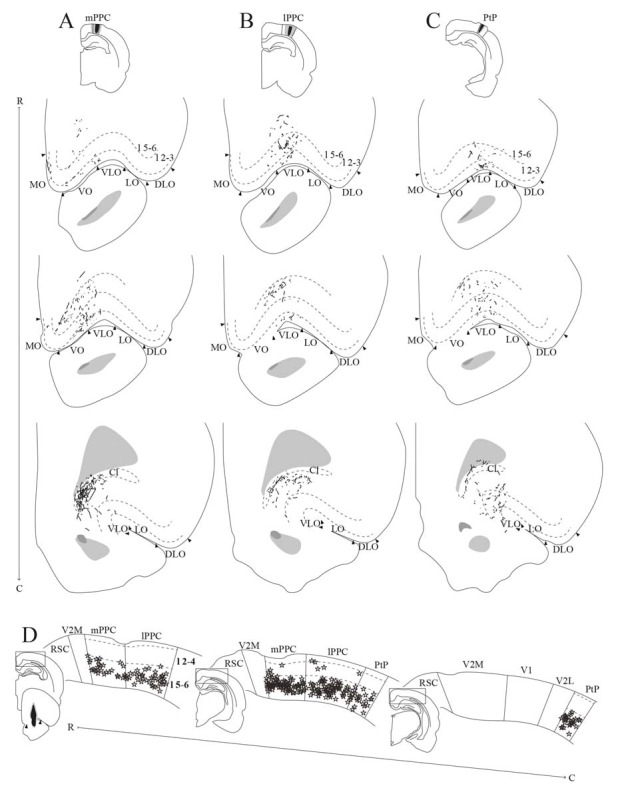
PPC projections to OFC. **(A–C)** Anterogradely labeled fibers at three different rostrocaudal levels (indicated by R–C) of OFC resulting from injections of PHA-L in mPPC **(A)**, lPPC **(B)**, and PtP **(C)**. As injections shifted from medial to lateral in PPC, anterogradely labeled fibers extended progressively more lateral and caudal in OFC. Arrowheads indicate borders between cortical areas and dashed lines demarcate cortical layers. Top insets indicate the position of the core of each injection with the borders of the mPPC **(A)**, lPPC **(B)**, and PtP **(C)** outlined with solid lines. **(D)** Retrogradely labeled cells represented by stars at three different rostrocaudal levels (indicated by R–C) of PPC resulting from a big injection of Fast Blue in the VO/VLO region. Labeled cells were primarily located in layer 5 across all PPC subdivisions. Solid lines indicate borders between cortical areas and dashed lines demarcate cortical layers. Bottom left inset shows a drawing of the injection site in a coronal section, arrowheads indicate the medial border of VO and lateral border of VLO. Insets at the left of each section indicate the rostrocaudal position of the depicted areas.

**Figure 11:**
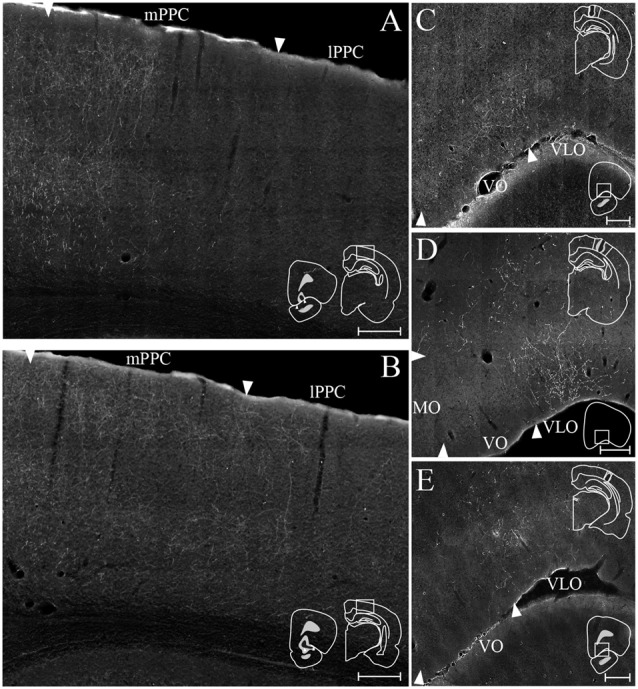
OFC-PPC connections. **(A,B)** Examples of labeled fibers in PPC resulting from injections of anterograde tracer in VO (**A**, BDA) and VLO (**B**, PHA-L). Insets indicate the position of the injection site in OFC with the borders of VO **(A)** and VLO **(B)** marked by arrowheads (left) and the portion of PPC containing the labeled fibers (right). **(C–E)** Examples of labeled fibers in OFC resulting from injections of anterograde tracer in mPPC (**C**, BDA), lPPC (**D**, PHA-L), and PtP (**E**, BDA). Insets indicate the position of the injection site in PPC with the borders of mPPC **(C)**, lPPC **(D)**, and PtP **(E)** outlined with solid lines (top) and the position in OFC of the depicted area (bottom). Arrowheads indicate borders of cortical areas. All scale bars: 200 μm.

#### VLO

Out of 13 injections of anterograde tracers in VLO, eight yielded anterogradely labeled fibers in PPC. From the cases with no labeled fibers in PPC, one injection was placed medially to the notch of the rhinal fissure, and the remaining four were placed lateral to the notch. A slight shift in the location of labeled fibers was observed between injections placed rostrally in VLO vs. injections situated at the more caudal portion of this area. A representative injection of PHA-L rostrally in VLO on the border with VO involved the medial bank of the rhinal fissure (Figure 9C). In mPPC, a moderately dense cluster of terminal fibers was observed, and more dispersed labeling was found in lPPC and PtP. Moderate to dense labeling was seen in V2M, whereas sparse labeling was encountered in V1 and V2L. Similar to what was seen in case of injections into VO, the majority of labeled fibers were located in layers 2 and 3, and labeling in layers 1 and 5 was somewhat sparser. An injection of BDA placed more caudally in VLO yielded sparse anterograde labeling in mPPC, lPPC, and PtP (Figure 9D). Only weak labeling was found in V2M, and a moderately dense plexus of labeled fibers was seen in V1. Terminating fibers were located mainly in layers 2 and 3, with sparser labeling in layers 1 and 5. A large injection of PHA-L placed caudally in VLO resulted in a moderately dense cluster of labeled fibers in mPPC and adjacent medial portions of lPPC, with sparser labeling more laterally in lPPC (Figure 11B) and PtP (not shown). Fibers tended to branch mainly in layers 2, 3, and 5.

To investigate the origin of orbitofrontal input to PPC, we reviewed the same dataset of retrograde tracers used to describe the projections from frontal midline areas to PPC, four cases of injections in mPPC and three cases of injections in lPPC (Figures 3G,P). All injections resulted in strong retrograde labeling in OFC, mainly in superficial layers. In one animal, Fluorogold was injected in mPPC and Fast Blue was injected in lPPC, with the cores of both injections located in superficial layers (Figure 9E). Both tracer injections resulted in dense labeling throughout the rostrocaudal extent of OFC. Cells labeled after the injection in mPPC spanned a substantial medial-to-lateral extent of OFC, from the medially located MO to the centrally located VLO. In contrast, the injection in lPPC yielded labeled cells mainly confined to VLO. Labeled cells from both injections were spatially intermingled, and occasional cells were found to be double-labeled. In view of the sparsity of these double-labeled neurons, we did not quantify these results. We observed very few labeled neurons in LO or DLO, in line with the results of anterograde tracers injected in either of the two areas.

### PPC Projections to Orbitofrontal Cortex

To analyze the distribution of PPC projections to OFC, we made use of the anterograde dataset with injections in PPC, described above (Figures 5–7G). Injections of anterograde tracers in PPC resulted in sparse to moderately dense terminal labeling in OFC with a distribution that matches that of retrogradely labeled neurons in OFC following injections in PPC, in that anterograde labeling was mainly found in MO, VO and VLO. Labeled fibers were largely observed in the hemisphere ipsilateral to the injection, although some fibers were encountered contralaterally in cases with strong orbitofrontal projections, terminating mainly in layer 1.

#### mPPC

In a representative case, PHA-L was injected rostrally in mPPC (Figure 10A). Anterogradely labeled fibers were observed in MO, VO and medially in VLO, across layers. At rostral levels of OFC, terminal fibers were mostly located in superficial layers, and at the most caudal level labeled fibers were seen to extend from the claustrum into VLO. Following an injection of BDA caudally in mPPC (Figure 11C), terminating fibers were mainly seen in the lateral portion of VO, extending into the medial portion of VLO. Fibers branched most densely in layer 3 but also extended into layers 2 and 1.

#### lPPC

An injection of PHA-L laterally in lPPC at a mid rostrocaudal position resulted in labeling largely confined to the medial half of VLO with a few fibers extending into the medially adjacent VO (Figure 10B). Terminating fibers were located mainly in layers 1, 3 and 6 throughout the rostrocaudal extent of VLO. Figure 11D shows labeled fibers after injection of PHA-L in lPPC in another case. A moderately dense cluster of terminal fibers was confined to the part of VLO located on the medial bank of the rhinal fissure. The fibers were seen to branch most densely in layer 1, and labeled fibers were scarcer in layers 2, 3 and 6. A few labeled fibers were also seen in deep layers of VO.

#### PtP

Similar to cases with injections in lPPC, injections of anterograde tracers in PtP yielded anterogradely labeled fibers mainly in VLO. In a representative case, PHA-L was injected rostrally and medially in PtP, slightly impinging on V2L (Figure 10C). The resulting labeled fibers were located mainly within VLO, with a slightly more lateral position in VLO than was seen following injections in lPPC. The labeled fibers were observed not only on the medial bank and notch of the rhinal fissure but extended into the lateral bank of the fissure as well. Terminating fibers were seen in layers 1–3, and 5/6. In another case, BDA was injected at a more caudal level of PtP (Figure 11E). As was seen following the more rostral injection, anterogradely labeled fibers, in this case, were mainly seen in medial VLO, with terminal branching largely in superficial layers.

In order to investigate the origin of PPC projections to OFC, retrograde tracers were injected in OFC in five cases. In a representative case, a large injection of Fast Blue was placed in the VO/VLO region, medial to the notch of the rhinal fissure (Figure 10D). Tracer leaked into the claustrum and the anterior olfactory nucleus, the latter was shown in our anterograde data to not receive projections from PPC. Across all subdivisions of PPC, retrogradely labeled cells were found in large numbers in deep layers and only occasionally were labeled cells found in superficial layers. In line with the sparse contralateral PPC-OFC projections observed in the anterograde tracer cases, very few labeled cells were observed in the contralateral PPC following injection of retrograde tracer in OFC (not illustrated).

## Discussion

**Figure 12:**
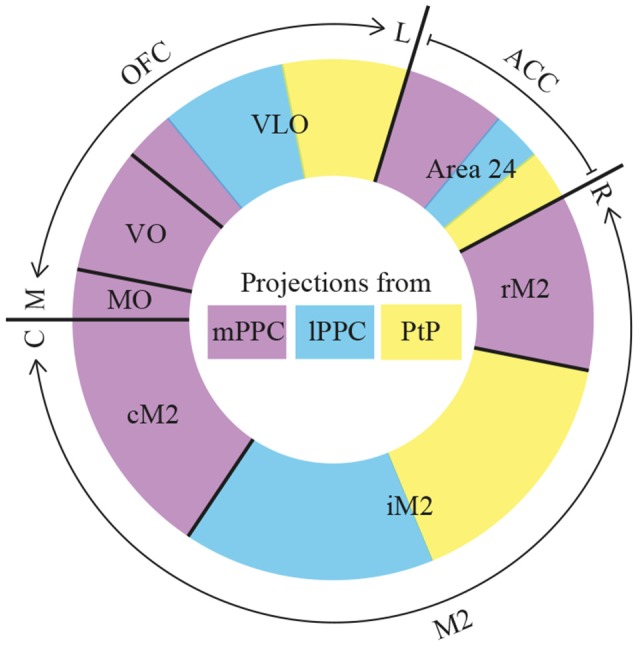
Summary. Diagram of parietal-frontal projections. The center of the circle shows a color-coded representation of the three PPC subdivisions, mPPC (purple), lPPC (blue), and PtP (yellow). The outer colored circle represents the relative proportion of projections, indicated by the size of the color-coded areas, from the three PPC subdivisions to frontal cortical areas that are separated with black lines. The outer edge indicates the total extent of orbitofrontal cortex (OFC), anterior cingulate cortex (ACC) and secondary motor cortex (M2), as well as their rostrocaudal (R–C) or mediolateral (M–L) organization. The reciprocal projections from OFC and M2 to PPC show comparable patterns. rM2, rostral portion of M2; iM2, intermediate portion of M2; cM2, caudal portion of M2.

The current data show that PPC projects to several frontal cortical areas (summarized in Figure 12), largely reciprocating the densest input received from the same areas. The laminar connection patterns are summarized in Supplementary Figure S3. Within the frontal midline areas, all PPC subdivisions appear to be strongly connected with M2. mPPC preferentially targets cM2 in addition to moderate labeling at rM2, whereas lPPC and PtP target iM2. Sparser connections were found with area 24b. mPPC projects to rostral 24b, whereas lPPC and PtP project to caudal regions. There were virtually no connections with area 24a, IL or PL. Within OFC, a gradient was revealed in which medial areas of OFC connects with mPPC, and the more central portion of OFC preferentially connects with lateral PPC subdivisions lPPC and PtP. PPC connections with the more lateral portions LO and DLO were absent. Our results are overall in line with previous studies, but as detailed below, our data uncover a topographical organization of the connections of the three subdivisions of PPC.

### Connections of PPC With Frontal Midline Cortices

#### Area M2

The current data corroborate previous reports that M2 projects to PPC, and that these projections originate from the entire rostrocaudal extent of M2 (Kolb and Walkey, 1987; Reep et al., 1994). Regarding the laminar origin of the M2 to PPC projections, we conclude that there is a slight preference for a deep origin, which seems disparate from what can be seen in the figures in the study of Reep et al. (1994), showing a more even laminar origin, although this is not specifically mentioned by the authors. The difference may be a result of the lower detail provided in the latter study compared to ours. In addition, our data showed that M2 projections to PPC originated in superficial layers at specific rostrocaudal levels. Projections from M2 to PPC have previously been shown to travel through layer 6 of the cortex (Reep et al., 1987), which is confirmed by our observations after injections of anterograde tracer in M2. Further, our data show that these projections terminate in layers 1 and 6 across mPPC and lPPC, with sparse, more diffuse labeling in PtP although, interestingly, the bilaminar labeling sometimes extends throughout all layers in a columnar pattern in mPPC and lPPC. Such an alternating bilaminar and columnar termination pattern of cortical projections of M2 has been described previously (Reep et al., 1987). With respect to possible connectional differences between the three subdivisions of PPC, the present data indicate that M2 projections to PtP are weak, whereas lPPC and mPPC both receive dense projections from M2. This differs from another study (Wilber et al., 2015), which reported that lPPC received much stronger M2 input than mPPC. A close evaluation of the latter study revealed that only one out of five retrograde tracer injections in lPPC showed particularly dense input from M2, one showed sparse input and the other three appeared to receive very little input. We consider it likely that the results of the one case showing strong labeling in M2 are due to the involvement of layer 6 in the injection site, since, as mentioned above, M2 projections to adjacent cortical areas have a preferred path through layer 6.

To our knowledge, we are the first to describe the laminar termination pattern of PPC projections within M2 and potential differences between projections from the three PPC subdivisions, although projections from PPC to M2 have been described in previous studies following injections of retrograde tracers in M2 in rats (Reep et al., 1984, 1990; Kolb and Walkey, 1987; Condé et al., 1995; Hoover and Vertes, 2007). These studies showed that the projections originated in layers 2/3 and 5 of PPC, however, one study found that projections to rM2 arose from deep layers only (Hoover and Vertes, 2007). Unfortunately, these studies provided little detail regarding the question of whether the three subdivisions of PPC contributed differentially to these projections, or whether topographical differences might be present. Moreover, detailed comparisons between the various studies are difficult since applied cortical delineations differ between studies, including our previous study in which we established the borders and subdivisions of PPC used here (Olsen and Witter, 2016). In mice, it was shown that extrastriate areas corresponding to PPC (anterior parts of A, AM and RL) also project to M2 (Wang et al., 2012) and, that the connectivity is reciprocal (Zingg et al., 2014; Hovde et al., 2018). The present results show that all PPC subdivisions project to superficial and deep layers of M2. In a previous study, it was reported that projections from nearby higher-order visual areas terminate in superficial layers of M2 (Miller and Vogt, 1984), but the authors did not include data on PPC projections.

As mentioned previously, M2 has been divided into three parts along its rostrocaudal axis. Injections of retrograde tracers showed that the rostral part of M2, rM2, received extensive somatosensory input whereas the caudal part, cM2, had an overweight of visual cortical input and the mid-portion, iM2, received a mixture of both (Reep et al., 1990). Our current data add to this by showing that PPC projections terminate at specific rostrocaudal levels within M2 as well. We further show that the terminal distribution of the PPC-M2 projection overlaps with the main origin of the reciprocating M2 projections to PPC. Thus, mPPC has minor reciprocal connections with a small portion at the rostral extreme of rM2, moderate reciprocal connections with the mid-portion and strongest reciprocal connections with the most caudal portion, cM2. lPPC is also sparsely reciprocally connected with rM2, but at a level much closer to the genu of the corpus callosum. In addition, strong reciprocal connections were found between lPPC and iM2, as well as a sparser connection with cM2. While we did not obtain any retrograde tracer injections confined to PtP, considering that PtP projects mainly to iM2, we expect that the densest input from iM2 to PtP originates here. However, it should be noted that, in our hands, three anterograde tracer injections in M2, located at different rostrocaudal levels of iM2 and cM2, yielded only sparse labeling in PtP compared to mPPC and lPPC.

#### PL, IL and Area 24

Our observations indicate that reciprocal connections between PPC and the prefrontal midline areas PL and IL are very sparse, in line with previous studies (Reep et al., 1994; Condé et al., 1995; Vertes, 2004; Hoover and Vertes, 2007; Wilber et al., 2015). Differing from the sparse or non-existent IL and PL connections, our data indicate that PPC has slightly stronger connections with ACC area 24, supporting previous reports (Condé et al., 1995; Hoover and Vertes, 2007). According to these studies, area 24 has corticocortical connections that overall are sparser than those of M2. Moreover, within ACC, area 24b has more widespread corticocortical connections than 24a (Vogt and Miller, 1983). Both notions are supported by our results.

Interconnections have been found between the IL and PL as well as the most rostral portion of area 24b (Condé et al., 1995; Fisk and Wyss, 1999; Jones et al., 2005; but see Vertes, 2004). These areas are only sparsely connected with the rest of the ACC as well as other cortical areas and are thought to constitute the medial prefrontal cortex in the rat. The mid- and caudal portions of area 24b are reciprocally connected with rostral and caudal area 24a in a complex manner (Jones et al., 2005), and receive input from several cortical areas (Reep et al., 1990). Data on connections between PPC and area 24 do not emphasize a particular preference to connect with any of these three rostrocaudal subdivisions. One might expect a relatively weak connection with the very rostral part of area 24, in line with the weak to absent connections with the connected PL and IL. However, our data suggest that mPPC receives input from the entire rostrocaudal extent of area 24b and has a small projection to the rostral extreme of area 24b as well as a very sparse projection to the most caudal portion that has been shown to originate mainly in deep layers (Finch et al., 1984; Condé et al., 1995; Hoover and Vertes, 2007). Regarding lPPC, it receives input only from the most caudal level of area 24b, and sends a minor reciprocating projection. The data on area 24 projections to PPC reported by us are in line with previously published ones, showing that injections of retrograde tracers in mPPC resulted in labeled cells throughout area 24b, and injections in lPPC yielded labeled cells confined to the caudal third of area 24b (Reep et al., 1994; see also Kolb and Walkey, 1987). PtP has a small projection to the caudal extreme of area 24b and currently, no data are available addressing a potential reciprocating projection in rodents. Thus, among the PPC subdivisions, mPPC appears to have the strongest connections with the ACC, including unique connections with the most rostral part.

### Connections of PPC With Orbitofrontal Cortex

Our data suggest that PPC is reciprocally connected with OFC, specifically to parts of OFC located medial to the orbital notch, in line with previous studies in rats and mice (Kolb and Walkey, 1987; Reep et al., 1994; Hoover and Vertes, 2011; Zingg et al., 2014; Wilber et al., 2015; Hovde et al., 2018). Retrograde tracer injections in PPC yielded retrogradely labeled cells largely in superficial layers of MO, VO and VLO, but not in the more lateral parts, LO and DLO (Kolb and Walkey, 1987; Reep et al., 1994; Wilber et al., 2015). Labeled cells in these three subdivisions of OFC appeared to be dispersed throughout, irrespective of whether the injection was placed in mPPC or lPPC (see especially Reep et al., 1994 for details). This contrasts with our data, where injections of retrograde tracer in mPPC resulted in labeled cells that were most concentrated in VO but were observed also in neighboring MO and medial VLO, and injections in lPPC yielded labeled cells mainly confined to medial VLO. Corroborating the retrograde data, our anterograde tracer data suggest that MO projects sparsely to PPC since only one out of seven injections yielded scattered anterogradely labeled fibers in PPC. In contrast, VO and VLO project progressively more lateral and caudal within PPC as injection sites shift from VO through rostral VLO to caudal VLO. Our findings replicate the results of a previous study where only a few labeled fibers were observed in layer 6 of PPC after injection of PHA-L in MO, whereas injections in VO produced labeled fibers, albeit sparse, in superficial and deep layers of PPC (Hoover and Vertes, 2011). Unfortunately, these authors did not include injections of anterograde tracers in VLO.

The topography of projections from PPC to OFC have not been studied in detail previously. To our knowledge, only one study employing retrograde tracers in OFC showed that MO, VO and VLO subdivisions received input from PPC, whereas LO did not (Reep et al., 1996). Our anterograde tracing data corroborate these observations in showing that PPC projections are confined to MO, VO and medial parts of VLO, with a strong preference for VO and medial VLO. Moreover, in line with the figures shown in the article by Reep et al. (1996), we showed that projections to VO and MO originate mainly from mPPC, whereas lPPC and PtP project almost exclusively to medial and central parts of VLO.

### Interconnectivity Between Frontal Midline and Orbital Cortices

Areas M2 and the medial half of OFC not only have reciprocal connections with PPC, they are also interconnected with each other. In particular, the rostrocaudal extent of M2 receives projections from superficial and deep layers of MO, VO, and VLO (Reep et al., 1984, 1990; Condé et al., 1995; Hoover and Vertes, 2007) that mainly terminate in layers 1 and 6 of M2 (Hoover and Vertes, 2011). Reciprocating projections from M2 to OFC target the same medial parts of OFC with preferred termination in layers 1, 3 and 5 of VLO, whereas only sparse projections were observed to LO (Reep et al., 1987). The preferred M2 projection to VLO/VO over MO or LO/DLO was corroborated by a retrograde study which showed that the projection originates from neurons in preferentially superficial but also deep layers of M2 along its rostrocaudal extent (Reep et al., 1996). In conclusion, M2 is reciprocally connected with the same portion of OFC as PPC is.

Whether or not connections between area 24 and OFC exist has been debated. One retrograde tracing study failed to observe projections from OFC to caudal parts of area 24a (Reep et al., 1990), whereas other studies have found projections to rostral area 24b that originate in MO, VO, and VLO (Condé et al., 1995; Hoover and Vertes, 2007). The first, negative report likely is caused by the fact that connections between OFC and area 24a, particularly its caudal portions, are overall very sparse (Hoover and Vertes, 2011).

Retrograde tracing studies consistently show that sparse reciprocal connections between area 24b and M2 exist, with cells in superficial and deep layers of both areas projecting to its neighbor, but details on their rostrocaudal topographical organization are not known (Reep et al., 1984, 1990; Condé et al., 1995; Hoover and Vertes, 2007). In an anterograde tracing study, projections from rostral M2 to area 24b were shown terminating sparsely across layers of 24b (Reep et al., 1987). However, results should be interpreted with caution since the two areas are adjacent to each other, and tracer injections in one area are at risk of leaking into the other.

### Functional Considerations

As mentioned previously, the focus of functional studies in the rodent PPC, along with frontal cortical areas, has shifted towards roles played by these areas in action control (Whitlock et al., 2008; Erlich et al., 2011; Harvey et al., 2012; Raposo et al., 2014), evidence accumulation (Hanks et al., 2015) and decision making (Raposo et al., 2014; Erlich et al., 2015). In the monkey, numerous anatomical and functional studies have suggested that a coarse somatotopy exists in sensory and motor responses within PPC, such that visual representations are located caudomedially, largely in area 7a, whereas somatic representations are dominant more rostrolaterally, in area 7b (Hyvärinen, 1981; Andersen et al., 1987; Rozzi et al., 2008). Such an organization is also reflected in the connections of areas 7a and 7b with respectively visual and somatosensory cortical areas (Cavada and Goldman-Rakic, 1989a; Andersen et al., 1990). Moreover, monkey PPC subdivisions are preferentially connected with different areas of the frontal cortex containing motor responses for the same part of the body (Cavada and Goldman-Rakic, 1989b; Andersen et al., 1990; Neal et al., 1990; Rozzi et al., 2006; Gharbawie et al., 2011; Stepniewska et al., 2011). The thalamic connections of the rat PPC suggest that mPPC may be homologous to monkey area 7a, whereas lPPC and PtP may be homologous to monkey area 7b (Olsen and Witter, 2016).

The results from the present study suggest that the three subdivisions of the rat PPC have topographically organized connections with frontal cortical areas which could be homologous to those of the monkey. Particularly dense connections exist with area M2, such that mPPC has strong reciprocal connections with cM2 and lPPC is reciprocally connected with iM2. Similarly, PtP projects to iM2 but details of reciprocal projections are still lacking. Within the rat M2, the distribution of somatosensory and visual input varies along its rostrocaudal axis (Reep et al., 1990), but whether the topography in somatic and visual sensory as well as posterior parietal connections translates into a functional topography remains unknown. Whisker, as well as eye motor responses, have been elicited by microstimulation of this area (Donoghue and Wise, 1982; Neafsey et al., 1986; Brecht et al., 2004), and the entire M2 has been suggested by some to be homologous to the primate frontal eye field (Neafsey et al., 1986; Reep et al., 1990), which is extensively interconnected with PPC. Another study examined the functional organization of motor cortex relative to input from S1, S2 and PPC (Smith and Alloway, 2013). By use of intracortical microstimulations, these authors found that motor cortex can be divided into a sensory processing zone and a motor-output area. Neurons in the transition zone between M1 and M2 were responsive to passive whisker deflections whereas M2 neurons were not. In contrast, the latter were more adequate at evoking whisker responses. Our results showed that lPPC and PtP partly target the transition zone in iM2, which would support their role in sensory integration and the homology to monkey area 7b.

Functional studies have also shown that activity in the rat M2 predicts upcoming movements (Erlich et al., 2011), as does activity in the rodent PPC (Whitlock et al., 2008; Harvey et al., 2012; Raposo et al., 2014), suggesting that the two areas are involved in similar functions. Interestingly, in a perceptual two-choice decision-making task, PPC activity reflected the upcoming action but was not essential for decision-making itself (Hanks et al., 2015). Rather, PPC neurons were constantly evaluating the sum of the presented stimulus. On the other hand, M2 activity was crucial for transforming the total value of the presented stimulus into a motor response (Erlich et al., 2015; Hanks et al., 2015). Further evidence for a functional relationship between PPC and M2 comes from a recent study in rats, which showed that both areas represent posture of the head and back and that spiking activity in PPC anticipates that of M2 (Mimica et al., 2018). Interestingly, the postural representations showed a strong topographical organization that appeared to correspond to the anatomical organization described in our study. Namely, the medial and rostral-most sectors of PPC and the caudal-most regions of M2 shared a predominant sensitivity to the posture of the animals’ backs, while the more lateral and caudal regions of PPC were dominated by signals for 3D head position, which was matched by similar tuning in mid-rostral coordinates in M2. This functional organization is specifically in line with the preferred connectivity of lPPC with iM2, and the strong connectivity of mPPC with cM2.

Whether ACC area 24b in the rat is a pure motor area may be debated, though microstimulation of neurons in this region elicits periocular, eye, and nose movements (Brecht et al., 2004). The most rostral part of area 24b is considered part of the medial prefrontal cortex based on its extensive connections with other medial frontal areas (Condé et al., 1995; Fisk and Wyss, 1999; Vertes, 2004; Jones et al., 2005), and our results suggest that this portion is connected exclusively with mPPC. This resembles the organization of monkey PPC-ACC connections where only area 7a receives area 24b input (Pandya et al., 1981). It should be mentioned that these authors described strong projections to area 7a as well as 7b from cingulate area 23, but a homolog of this area has thus far not been found in rodents.

Our data further indicate that the rat PPC is reciprocally connected with OFC medial to the orbital notch in a topographical manner. Anatomical studies have suggested that the connectivity of primate OFC subdivisions is organized in two distinct networks, a medial prefrontal and an orbital one, each of which subserves different functions, and these observations have been extended to the rat OFC (Floyd et al., 2000, 2001; Öngur and Price, 2000; Price, 2007). However, the interpretation of the functional relevance of these networks is confounded by the fact that several OFC subdivisions have connections within both networks. Nevertheless, it is interesting to note in our results that the rat PPC is connected with only a subset of OFC subdivisions located medial to the orbital notch, suggesting a functional specialization of this portion of OFC. It has been suggested that the lateral half of the OFC evaluates individual options for choosing behavior, whereas the medial half compares the choices according to their reward size and probability (for review, see Rudebeck and Murray, 2014). Functional studies in primates have indicated that (pre)frontal cortices, in general, exert a top-down control over PPC during goal-directed actions (Buschman and Miller, 2007; Crowe et al., 2013). Thus, OFC could inform PPC about the value of expected outcomes and perhaps influence the evaluation of appropriate actions within PPC, which would then guide the execution of actions along with motor regions. Even though PPC subdivisions and frontal cortical areas may be interconnected within the same networks, the topographical organization of connections between them found in the present study suggests that functional differences between PPC subdivisions exist. However, further work is required to establish precisely what these differences entail.

## Data Availability

All datasets generated for this study are included in the manuscript and/or the Supplementary Files.

## Ethics Statement

### Animal Subjects

The animal study was reviewed and approved by Norwegian Food Safety Authority Animal Welfare Committee of the Norwegian University of Science, and Technology European Communities Council Directive and the Norwegian animal welfare act.

## Author Contributions

All authors had full access to all the data in the study and take responsibility for the integrity of the data and the accuracy of the data analysis. MW: obtained funding, study supervision, study concept and design. GO, KH, HK, TS and HS: acquisition of data. GO, KH, TS, HS and MW: analysis and interpretation of the data. GO and KH: drafting of the manuscript. MW and JW: critical revision of the manuscript for important intellectual content.

Study concept and design: MPW. Acquisition of data: GO, KH, HK, TS, HS. Analysis and interpretation of the data: GO, KH, TS, HS, MW. Drafting of the manuscript: GO and KH. Critical revision of the manuscript for important intellectual content: MW, JW. Obtained funding: MW. Study supervision: MW.

## Conflict of Interest Statement

The authors declare that the research was conducted in the absence of any commercial or financial relationships that could be construed as a potential conflict of interest.

## Abbreviations: Brain areas:

24a: anterior cingulate area 24a
24b: anterior cingulate area 24b
ACC: anterior cingulate cortex
Cl: claustrum
cM2: caudal secondary motor cortex
DLO: dorsolateral orbitofrontal cortex
iM2: intermediate secondary motor cortex
IL: infralimbic cortex
Ins: insular cortex
LO: lateral orbitofrontal cortex
lPPC: lateral posterior parietal cortex
M1: primary motor cortex
M2: secondary motor cortex
MO: medial orbitofrontal cortex
mPPC: medial posterior parietal cortex
OFC: orbitofrontal cortex
PL: prelimbic cortex
PPC: posterior parietal cortex
PtP: caudolateral posterior parietal cortex
rM2: rostral secondary motor cortex
RSC: retrosplenial cortex
S1: primary somatosensory cortex
V1: primary visual cortex
V2: secondary visual cortex
V2L: lateral secondary visual cortex
V2M: medial secondary visual cortex
VLO: ventrolateral orbitofrontal cortex
VO: ventral orbitofrontal cortex.

## Others:

BDA: biotinylated Dextran amine
C: caudal
DAB: 3,3′-diaminobenzidine tetrahydrocholoride
DMSO: dimethylsulfoxide
FB: Fast Blue
FG: Fluorogold
L: lateral
M: medial
PHA-L: *Phaseolus vulgaris* agglutinin
R: rostral.
