# Supplementary material for: Organization of Posterior Parietal–Frontal Connections in the Rat
Source: Front Syst Neurosci. 2019 Aug 21;13:38. doi: 10.3389/fnsys.2019.00038 (PMC6713060; doi:10.3389/fnsys.2019.00038)
Supplement: Supplementary file 1 [file Data_Sheet_1.pdf]

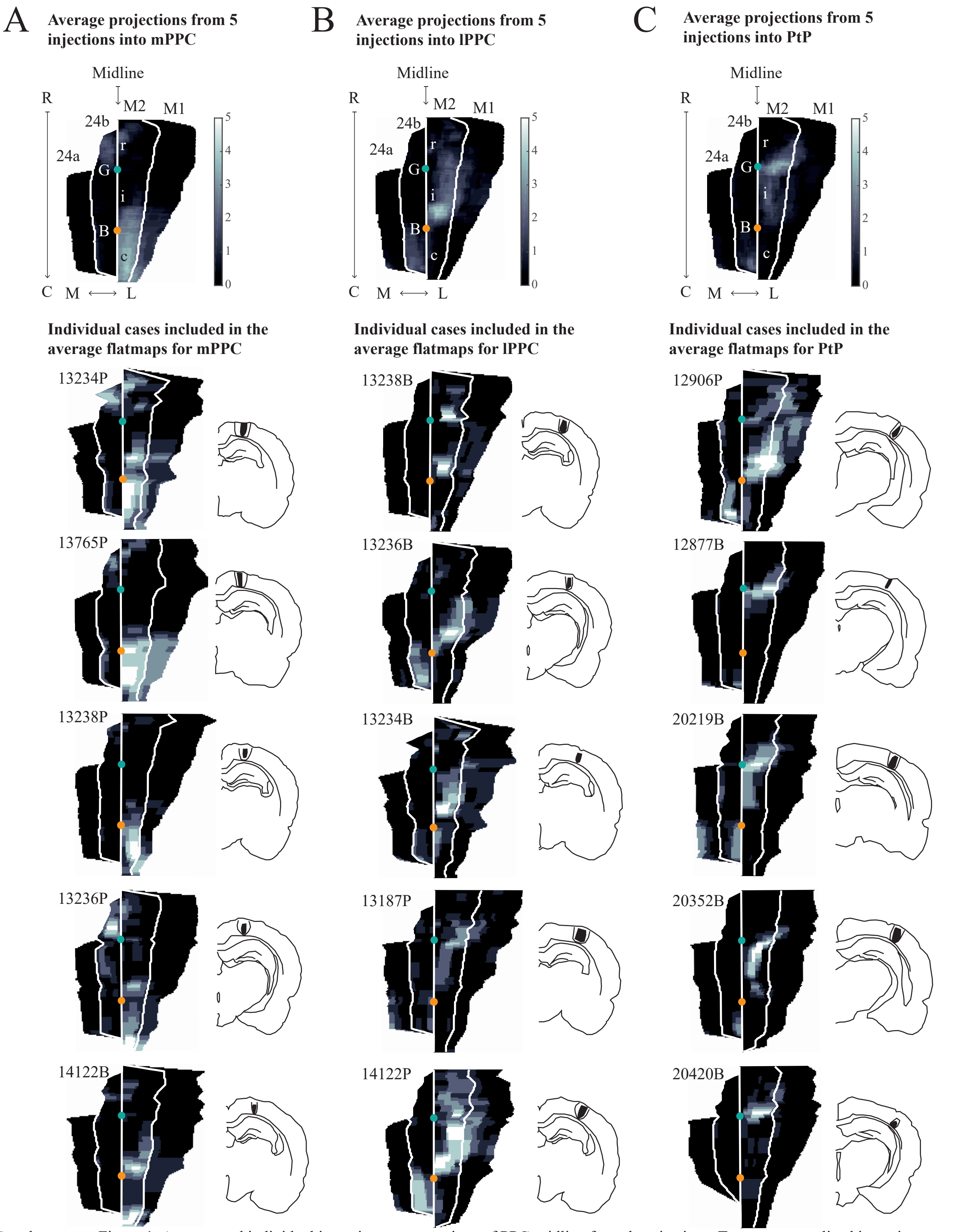

Supplementary Figure 1: Average and individual intensity representations of PPC-midline frontal projections. Top row, normalized intensity representation of the density of labeling in frontal midline areas averaged from five injections into mPPC **(A)**, lPPC **(B)** and PtP **(C)**, arranged from rostral to caudal (R – C); lighter colors indicate a denser plexus of labeled fibers and numbers indicate the density score. Bregma (B) is indicated by an orange circle and the genu of the corpus callosum (G) by a green circle. Below, each column contains the intensity representations of five individual cases of tracer injection in mPPC (left), lPPC (center), and PtP (right) that made the basis for the normalized representations. Indicated above each map is the animal's number and the tracer used and, to the right, the injection site of the tracers.
