# Supplementary material for: Organization of Posterior Parietal–Frontal Connections in the Rat
Source: Front Syst Neurosci. 2019 Aug 21;13:38. doi: 10.3389/fnsys.2019.00038 (PMC6713060; doi:10.3389/fnsys.2019.00038)
Supplement: Supplementary file 2 [file Data_Sheet_2.PDF]

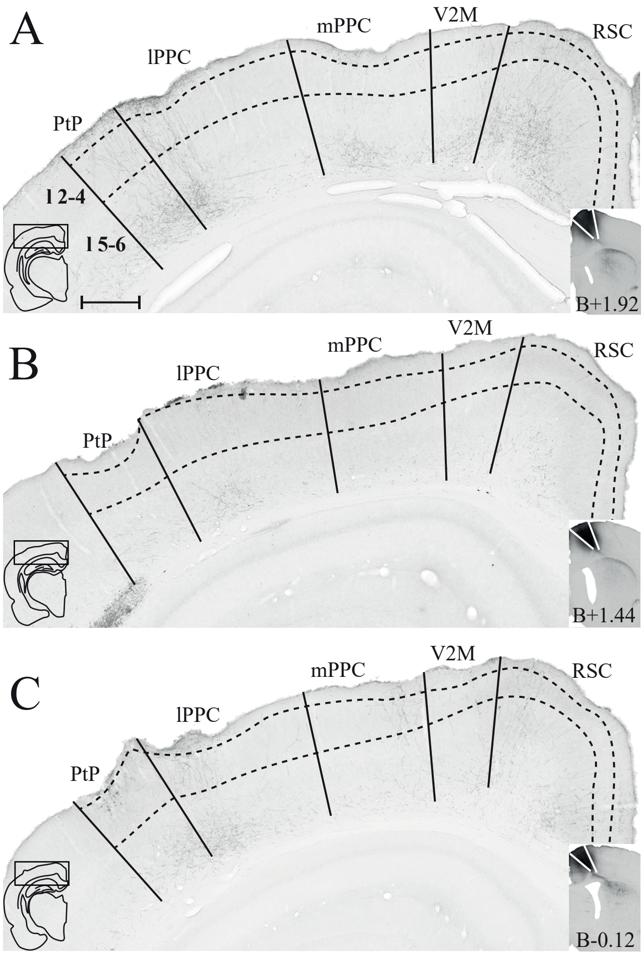

Supplementary Figure 2: M2 projections to contralateral PPC. Anterogradely labeled fibers in contralateral PPC and nearby areas resulting from three injections of PHA-L, at similar rostrocaudal levels where strong ipsilateral labeling was observed in the same cases (see Figure 4). For the most rostral injection (A), a few labeled fibers were seen in mPPC, lPPC and PtP, whereas the more caudal injections (B, C) resulted in the densest labeling in lPPC. Solid lines indicate borders between cortical areas and dashed lines demarcate cortical layers. Insets are images of the injection site cores, with the borders of M2 indicated by solid lines (right), and an outline of the hemisphere for each section with labeled fibers (left). Bregma levels are approximate and according to Paxinos and Watson (2007). Scalebar 500 µm.
