# Supplementary material for: Organization of Posterior Parietal–Frontal Connections in the Rat
Source: Front Syst Neurosci. 2019 Aug 21;13:38. doi: 10.3389/fnsys.2019.00038 (PMC6713060; doi:10.3389/fnsys.2019.00038)
Supplement: Supplementary file 3 [file Data_Sheet_3.PDF]

A

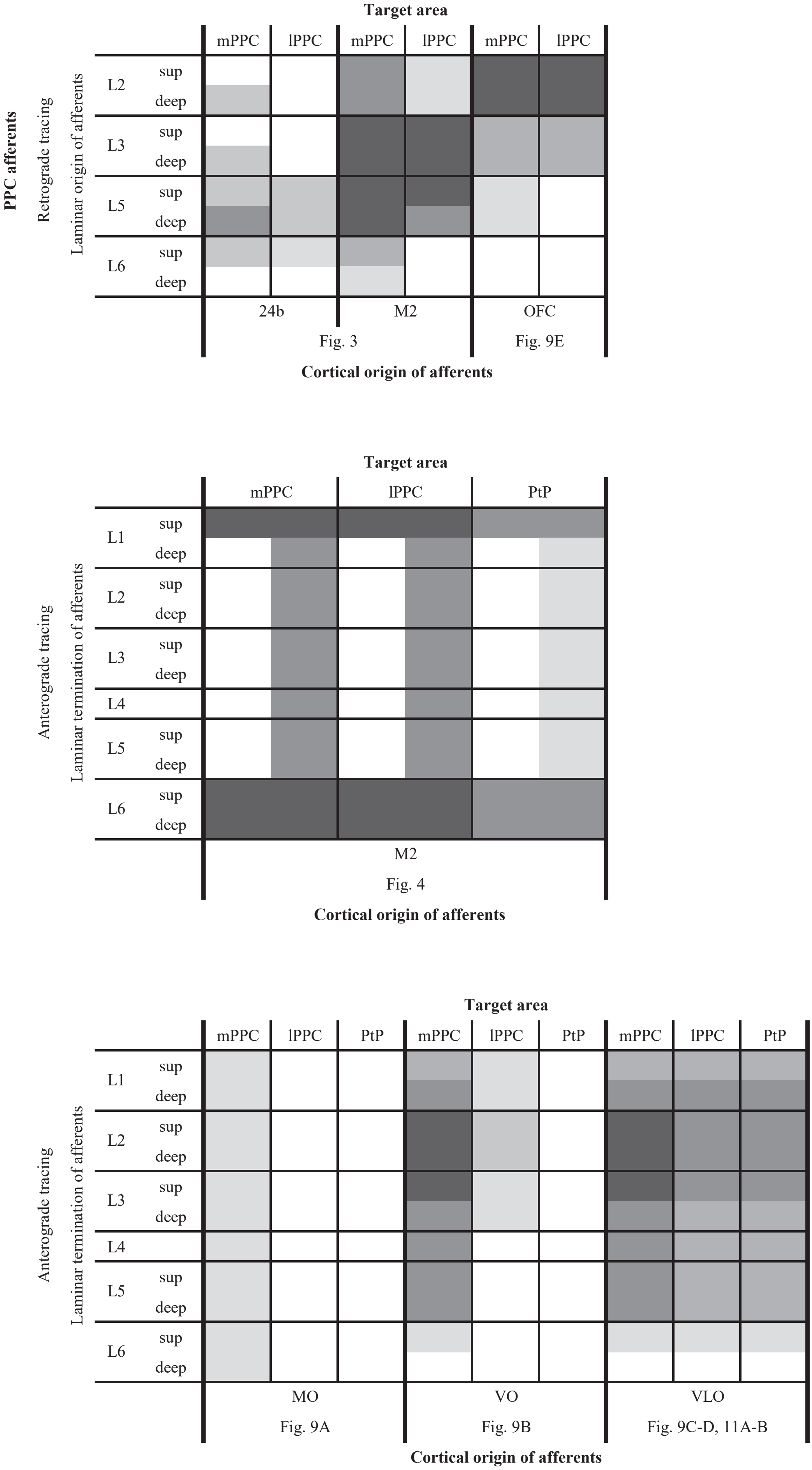
Target area
mPPC
lPPC
mPPC
lPPC
mPPC
lPPC
PPC afferents
Retrograde tracing
Laminar origin of afferents
L2
L3
L5
L6
sup
deep
24b
M2
OFC
Fig. 3
Fig. 9E
Cortical origin of afferents
Target area
mPPC
lPPC
PtP
Anterograde tracing
Laminar termination of afferents
L1
L2
L3
L4
L5
L6
sup
deep
M2
Fig. 4
Cortical origin of afferents
Target area
mPPC
lPPC
PtP
mPPC
lPPC
PtP
mPPC
lPPC
PtP
Anterograde tracing
Laminar termination of afferents
L1
L2
L3
L4
L5
L6
sup
deep
MO
VO
VLO
Fig. 9A
Fig. 9B
Fig. 9C-D, 11A-B
Cortical origin of afferents

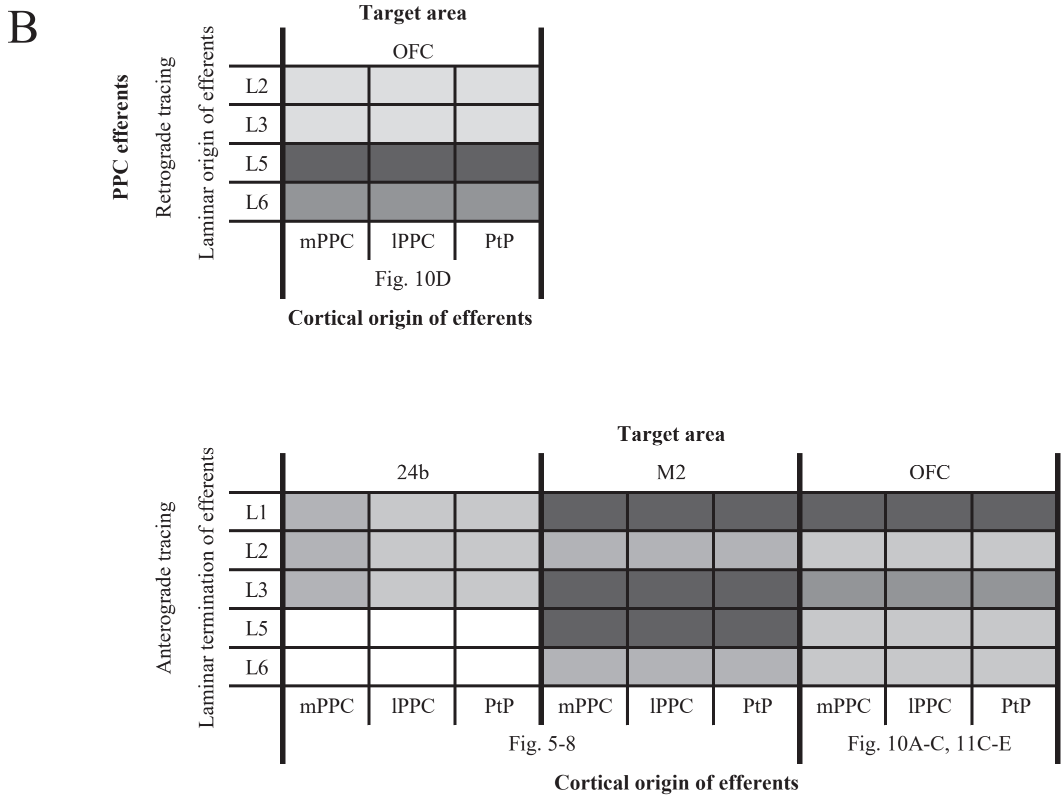

Supplementary Figure 3: Laminar pattern of PPC connections with frontal cortical areas. **A**, top, laminar origin of PPC afferents from 24b and M2 (from Figure 3) and from OFC (from Figure 9E). Middle, alternating bilaminar and columnar termination pattern of afferents to PPC from M2 (from Figure 4); bottom, laminar termination of afferents to PPC from MO, VO and VLO (from Figures 9A-D, 11A-B).
B, top, laminar origin of PPC efferents to OFC (from Figure 10D). Bottom, laminar termination of efferents from PPC to 24b, M2 and OFC (from Figures 5-8, Figure 10A-C, 11C-E).
